# Comparison of physical fitness in youth with post-COVID-19: A study of individuals with and without symptoms

**DOI:** 10.3934/publichealth.2025051

**Published:** 2025-10-22

**Authors:** Patchareeya Amput, Sirima Wongphon

**Affiliations:** 1 Department of Physical Therapy, School of Allied Health Sciences, University of Phayao, Phayao 56000, Thailand; 2 Unit of Excellence of Human Performance and Rehabilitations, University of Phayao, Phayao, Thailand, Phayao 56000, Thailand; 3 Department of Traditional Chinese Medicine, School of Public Health, University of Phayao, Phayao 56000, Thailand

**Keywords:** post-COVID-19, youth, muscle strength, respiratory system, physical fitness

## Abstract

This study aims to examine differences in physical fitness among young adults in three distinct groups: individuals with long COVID, those who had recovered from COVID-19 without lingering symptoms, and healthy individuals with no history of infection. A total of 105 participants were equally divided into the three groups (n = 35 each). Evaluations included handgrip strength for upper body strength, handheld dynamometry for quadricep strength, and a six-minute walk test (6MWT) to assess the cardiorespiratory performance. Participants with long COVID demonstrated significantly lower handgrip strengths compared to the control group. Additionally, both post-COVID groups showed reduced 6MWT distances and elevated post-exercise physiological responses, including heart rate, systolic blood pressure, perceived exertion, and leg fatigue, regardless of symptom persistence. These findings indicate that individuals recovering from COVID-19, especially those with persistent symptoms, exhibit measurable declines in muscular and cardiorespiratory fitness, along with heightened physiological stress during physical activity.

## 1. Introduction

Post-coronavirus disease 2019 (COVID-19) involves the persistence of symptoms long after the initial infection caused by the severe acute respiratory syndrome coronavirus 2 (SARS-CoV-2) virus, typically occurring three months after the onset of COVID-19. These individuals experience symptoms that persist for at least two months and cannot be explained by any alternative diagnosis [1]. Post-COVID-19 is characterized by lingering symptoms such as fatigue, dyspnea, muscle weakness, and a reduced exercise tolerance, all of which can significantly impair daily functioning and the overall quality of life [2], thus leading to limitations in social and physical activities [3],[4]. Additionally, several studies have reported that post-COVID-19 patients experience worsening symptoms with physical exertion, thus resulting in exercise intolerance [5],[6]. These conditions are associated with impaired oxygen uptake, autonomic function, and inflammation [7]–[9].

Physical fitness plays a crucial role in the overall health and functional independence, making it essential to evaluate whether post-COVID-19 patients experience long-term impairments in their physical performance. Several studies have reported declines in cardiorespiratory fitness, muscle strength, and endurance in individuals recovering from COVID-19, particularly those with long COVID [10]–[12]. These impairments may result from various physiological mechanisms, including persistent inflammation, autonomic dysfunction, mitochondrial abnormalities, and deconditioning due to prolonged illness or reduced physical activity during recovery [5]–[7]. Additionally, respiratory complications, such as a decreased lung diffusion capacity and impaired oxygen uptake, may further contribute to exercise intolerance in post-COVID-19 individuals. The combination of these factors can lead to fatigue, a reduced functional capacity, and limitations in performing physical activities, ultimately impacting long-term recovery and quality of life [13]. However, there is limited research that directly compares physical fitness between post-COVID-19 patients with and without persistent symptoms. Understanding these differences is crucial to design targeted rehabilitation strategies to enhance the recovery outcomes. To bridge these gaps in knowledge, this study aims to compare the physical fitness of post-COVID-19 patients with long COVID, those without persistent symptoms, and individuals who have never had a COVID-19 infection. The assessment includes measures of muscle strength and cardiorespiratory fitness. The findings of this study will provide valuable insights into the long-term effects of COVID-19 on physical health and inform rehabilitation approaches to enhance recovery and the quality of life of affected individuals.

## 2. Materials and methods

### 2.1. Study design

This cross-sectional investigation evaluated physical fitness across three young adult groups. Tests included handgrip dynamometry for upper body strength, handheld dynamometry for quadricep strength, and a six-minute walk test (6MWT) to assess the cardiorespiratory capacity.

### 2.2. Participants

The study included post-COVID-19 patients with long COVID, those without persistent symptoms, and individuals who have never had a COVID-19 infection. A total of 105 participants were recruited and evenly assigned to three groups (n = 35/group). The sample size was determined through a power analysis, with a statistical power of 0.95, an alpha level of 0.05, and an effect size d of 0.40 [14]. Three groups of age- and sex-matched adults were recruited: the control group (CG) included individuals who had never had a COVID-19 infection; the persistent symptom group (PSG) included individuals who experienced persistent symptoms for more than 12 weeks; and the recovered COVID group (RCG) consisted of individuals who had previously developed COVID-19 but recovered without persistent symptoms. The inclusion criterion required participants to be 18 years or older. Those in the long COVID and recovered groups had confirmed COVID-19 diagnoses via either PCR or antigen testing at least 12 weeks prior to the study. The long COVID group included participants with symptoms that lasted over 12 weeks, whereas the recovered group had resolved symptoms within four weeks post-infection. The control group members had no history of COVID-19. Individuals were excluded if they had a body mass index (BMI) over 30 kg/m², an oxygen saturation below 95%, or pre-existing conditions that affect the cardiopulmonary or musculoskeletal systems. All participants provided written informed consent after being fully informed about the study's protocol. Ethical clearance was granted by the University of Phayao Human Research Ethics Committee (HREC–UP–HSST 1.2/110/67).

### 2.3. Procedure

The participants completed interviews to collect demographic and medical data, including anthropometric measurements and vital signs. The physical assessments are described below.

*Handgrip strength test: Measured using a T.K.K 5001 Grip-A dynamometer* [15].

Each participant gripped the device with their dominant hand for three seconds while standing with the arm extended and slightly abducted. The test was performed three times, with one-minute rest intervals between trials, and the highest score (in kilograms) was recorded [16].

*Quadriceps muscle strength: Evaluated using a* hand-held dynamometer (Model-01165, Lafayette Instrument Company, Lafayette IN, USA) [17].

The participants were seated with their hips and knees flexed at 90 degrees and performed maximal isometric knee extensions using their dominant lower limb. The test was conducted at least three times, and the highest recorded value (in kilograms) was used for the analysis [18].

*6-minute walk test*.

Before the test, the participants rested in a seated position for at least 10 minutes to ensure they were adequately prepared. Then, baseline cardiorespiratory parameters, including heart rate (HR), systolic blood pressure (SBP), diastolic blood pressure (DBP), oxygen saturation (SpO_2_), rate of perceived exertion (RPE), and leg fatigue, were measured. The test was conducted along a 30-meter corridor, where the participants were instructed to walk as far as possible within a 6-minute period. Running was not permitted, but the participants were encouraged to walk at a comfortable pace, with the option to slow down or take a brief rest if needed. The total distance covered during the 6MWT was recorded to assess the participant's cardiorespiratory fitness [19]. After the test, the cardiorespiratory parameters were measured again.

### 2.4. Statistical analysis

Descriptive statistics were calculated for all variables. A one-way ANOVA was used to identify group differences in muscular strength and physiological responses before and after the 6MWT. The statistical significance was set at p < 0.05. Analyses were carried out using the SPSS software, version 22.0.

## 3. Results

The characteristics of the participants are shown in Table 1. There were no statistically significant differences in age, weight, height, or BMI among the groups. The average time since the first COVID-19 infection was 7.51 months for the PSG and 7.31 months for the RCG. The average number of vaccine doses received was 2.80 for the CG, 2.89 for the PSC, and 2.71 for the RCG.

**Table 1.** Characteristics of the participants. Values are presented as means ± SD.

**Variables**	**CG** **(n = 35; F = 20, M = 15)**	**PSG** **(n = 35; F = 20, M = 15)**	**RCG** **(n = 35; F = 20, M = 15)**
Age (years)	20.14 ± 0.65	20.23 ± 0.12	20.20 ± 0.13
Weight (kg)	60.80 ± 1.43	60.86 ± 1.54	59.20 ± 1.56
Height (m)	1.66 ± 0.01	1.67 ± 0.01	1.67 ± 0.01
BMI (kg/m^2^)	21.79 ± 0.33	21.67 ± 0.37	21.07 ± 0.34
Time since first COVID-19 infection, month	-	7.51	7.31
Vaccination status, numbers	2.8	2.89	2.71

Note: CG = control group; PSG = persistent symptom group; RCG = recovered COVID group; F = female; M = male; BMI = body mass index.

The handgrip strength test showed a significant decrease for the PSG compared to the CG (p < 0.05). There were no statistically significant differences in quadricep strength among the groups. The 6MWT distance was significantly decreased for the PSG and RCG compared to the CG (Table 2).

**Table 2.** Comparison of upper and lower limb muscle strength and 6MWT distance among groups.

Variables	CG	PSG	RCG
Handgrip strength test (kg)	33.35 ± 5.14	30.50 ± 6.09^a^	33.03 ± 6.47
Quadricep strength (kg)	24.53 ± 2.16	23.56 ± 3.26	24.74 ± 4.98
Distance of the 6MWT	601.29 ± 30.27	588.71 ± 16.85^a^	590.14 ± 18.09^a^

Note: CG = control group; PSG = persistent symptom group; RCG = recovered COVID group; 6MWT = 6-minute walk test; ^a^p < 0.05 vs. CG and ^b^p < 0.05 vs. PSG.

The baseline cardiorespiratory parameters showed no statistically significant differences among the groups. The post-HR, post-SBP, post-RPE, and post-leg fatigue were significantly increased for the PSG and RCG compared to the CG (p < 0.05) after performing the 6WMT (Table 3).

**Table 3.** Comparison of cardiorespiratory parameters from the 6MWT among groups.

**Variables**	**CG**	**PSG**	**RCG**
Pre-HR (bpm)	80.40 ± 9.18	82.00 ± 7.30	80.80 ± 8.86
Post-HR (bpm)	114.77 ± 9.29	118.69 ± 7.10^a^	120.74 ± 7.74^a^
Pre-SBP (mmHg)	118.49 ± 11.36	120.23 ± 9.74	118.23 ± 11.32
Post-SBP (mmHg)	136.43 ± 9.58	142.17 ± 6.02^a^	141.11 ± 9.25^a^
Pre-DBP (mmHg)	78.71 ± 7.83	77.00 ± 6.22	77.29 ± 7.33
Post-DBP (mmHg)	78.94 ± 6.41	79.31 ± 6.01	78.43 ± 6.35
Pre-O_2_ sat (%)	98.14 ± 0.69	98.43 ± 0.70	98.31 ± 0.63
Post-O_2_ sat (%)	97.40 ± 0.65	97.31 ± 0.47	97.40 ± 0.50
Post-RPE	9.26 ± 1.22	11.09 ± 1.40^a^	10.74 ± 1.24^a^
Post-leg fatigue	1.77 ± 0.77	2.49 ± 0.78^a^	2.43 ± 0.81^a^

Note: CG = control group; PSG = persistent symptom group; RCG = recovered COVID group; HR = heart rate; SBP = systolic blood pressure; DBP = diastolic blood pressure; O_2_ sat = pulse oxygen saturation; RPE = rate of perceived exertion; ^a^p < 0.05 vs. CG and ^b^p < 0.05 vs. PSG.

## 4. Discussion

This study indicates that individuals who experienced persistent symptoms for more than 12 weeks had decreased upper limb muscle strengths compared to healthy individuals. Additionally, both individuals with persistent symptoms for more than 12 weeks and those who had recovered from COVID-19 without persistent symptoms showed decreased 6MWT distances and increased post-HR, post-SBP, post-RPE, and post-leg fatigue when compared to healthy individuals. The findings reveal significant impairments in muscle strength and cardiorespiratory fitness, particularly among individuals who experienced long COVID.

Our results indicate that the upper limb muscle strength, assessed through the hand-grip strength test, was significantly reduced for the PSG compared to the CG. This decline in strength is consistent with previous studies that reported muscle weakness in individuals with long COVID, which may be attributed to persistent inflammation, mitochondrial dysfunction, and physical inactivity during the recovery phase [12],[20]–[23]. These findings are consistent with prior studies and highlight the importance of monitoring muscle strength in post-COVID-19 rehabilitation. Persistent inflammation has been widely recognized as a key factor that contributes to muscle weakness in long COVID patients. Studies suggest that elevated levels of pro-inflammatory cytokines, such as interleukin-6 (IL-6) and tumor necrosis factor-alpha (TNF-α), may promote muscle catabolism, reduce protein synthesis, and impair muscle regeneration [24]. This chronic inflammatory state may explain the observed reduction in upper limb muscle strength, particularly in individuals who experienced prolonged post-COVID symptoms. Additionally, mitochondrial dysfunction has been implicated in post-viral fatigue and muscle weakness. Research has shown that SARS-CoV-2 can disrupt mitochondrial function, thus leading to impaired energy production, increased oxidative stress, and muscle fatigue [25],[26]. These mitochondrial abnormalities may contribute to the decline in handgrip strength for+ the PSG, as muscle contraction and endurance heavily depend on mitochondrial ATP production. In contrast, quadricep strength did not significantly differ among the groups. While some post-COVID-19 patients have exhibited lower limb muscle weakness, the absence of a statistically significant difference in this study suggests that the extent of lower limb impairment may depend on factors such as disease severity, activity level, or recovery duration [22],[23]. One possible explanation for this finding is that daily activities naturally involve lower limb movement, such as walking, standing, and stair climbing, which could help maintain muscle strength. In contrast, the upper limb muscles are less frequently engaged in routine activities, thus potentially leading to a more noticeable decline in strength [18]. Although some results, such as quadricep strength (PSG mean 23.56 kg vs. CG 24.53 kg), did not reach statistical significance, we acknowledge that exploring trends in these measures and conducting subgroup analyses (e.g., by sex) could provide additional insights. The upper limb muscle weakness observed for the PSG aligns with previous studies that reported post-COVID-19 muscle impairment [10],[18]–[21], whereas the absence of significant lower limb differences may reflect variations in the study populations, disease severity, physical activity, or recovery duration. Additionally, we note that our participants were primarily young to middle-aged adults, and findings may not fully generalize to older populations [8]–[10].

The 6MWT distance was significantly reduced for both the PSG and RCG compared to the CG, thus indicating a diminished functional capacity, even in individuals who had recovered without persistent symptoms. However, limitations include a lack of gas exchange measurements, autonomic function tests, and pre-morbid physical activity data. This finding is consistent with prior studies that demonstrated a reduced exercise tolerance among post-COVID-19 patients, which may be attributed to the lingering effects on pulmonary function, cardiovascular health, or muscle metabolism [5]–[7]. The more pronounced reductions in walking distance observed for the PSG suggests that long COVID further exacerbates the physical limitations, potentially due to sustained inflammation, autonomic dysfunction, or prolonged deconditioning [12]. Furthermore, the cardiorespiratory responses during the 6MWT revealed increased post-exercise HR, SBP, RPE, and leg fatigue for both the PSG and RCG compared to the CG. These findings indicate that post-COVID-19 individuals experience greater physiological strain during physical activity, which may stem from autonomic dysregulation, impaired oxygen uptake, or cardiovascular deconditioning [7]. The heightened exertion and fatigue responses for the PSG further reinforce the notion that persistent symptoms contribute to prolonged impairments in physical fitness and exercise tolerance.

## 5. Strength and limitations of this study

This study has several limitations that should be acknowledged. The present study demonstrated no significant differences among groups in age, weight, height, BMI, vaccination status, or the average time since the first COVID-19 infection, thus indicating that participants were generally well matched. However, the severity of prior COVID-19 infection (e.g., hospitalization, oxygen supplementation) was not assessed. This limitation may have influenced the recovery trajectories and should be considered when interpreting the findings. In addition, the physical activity levels before and after COVID-19 infection were not measured, which could potentially explain some degree of deconditioning. Finally, the interpretation of our findings relies on between-group comparisons without individual pre-morbid fitness data. Overall, these findings highlight the importance of monitoring muscle strength and functional capacity in post-COVID-19 rehabilitation, as well as provide valuable insights for designing targeted interventions to improve long-term recovery and the quality of life.

## 6. Conclusions

Persistent symptoms following COVID-19, particularly in individuals with long COVID, lead to significant reductions in the upper limb muscle strength and functional exercise capacity, even for those who have recovered from acute infection. The study highlights the physiological strain experienced during physical activity and underscores the importance of monitoring and implementing targeted rehabilitation interventions. Our findings provide critical evidence to inform strategies aimed at improving long-term recovery and the quality of life of post-COVID-19 patients.

## Use of AI tools declaration

The authors declare they have not used Artificial Intelligence (AI) tools in the creation of this article.
